# Cloning and Expression Analysis of the *Bombyx mori α-amylase* Gene (*Amy*) from the Indigenous Thai Silkworm Strain, Nanglai

**DOI:** 10.1673/031.011.0138

**Published:** 2011-03-29

**Authors:** Nipaporn Ngernyuang, Isao Kobayashi, Amornrat Promboon, Sunanta Ratanapo, Toshiki Tamura, Lertluk Ngernsiri

**Affiliations:** ^1^ The Department of Genetics, Faculty of Science, Kasetsart University, Bangkok, Thailand; ^2^ The National Institute of Agrobiological Sciences, Tsukuba, Ibaraki 305-8634, Japan; ^3^ The Department of Biochemistry, Faculty of Science, Kasetsart University, Bangkok, Thailand; ^4^ These two authors contributed equally to this work.

**Keywords:** *Amy*, *Bombyx mori*, *GAL4-UAS*, silkworm

## Abstract

α-Amylase is a common enzyme for hydrolyzing starch. In the silkworm, *Bombyx mori* L. (Lepidoptera: Bombycidae), α-amylase is found in both digestive fluid and hemolymph. Here, the complete genomic sequence of the *Amy* gene encoding α-amylase from a local Thai silkworm, the Nanglai strain, was obtained. This gene was 7981 bp long with 9 exons. The full length *Amy* cDNA sequence was 1749 bp containing a 1503 bp open reading frame. The ORF encoded 500 amino acid residues. The deduced protein showed 81–54% identity to other insect α-amylases and more than 50% identity to mammalian enzymes. Southern blot analysis revealed that in the Nanglai strain *Amy* is a single-copy gene. RT- PCR showed that *Amy* was transcribed only in the foregut. Transgenic *B. mori* also showed that the *Amy* promoter activates expression of the transgene only in the foregut.

## Introduction

α-Amylase (α-1,4-glucan-4-glucanohydrolase) is a member of the glycoside hydrolase family 13, that catalyzes the hydrolysis of the α-(1,4) glycosidic linkages in starch and related compounds (Janecek 1997). α-Amylases from many animals have been characterized both biochemically and molecularly. In the silkworm, *Bombyx mori* L. (Lepidoptera: Bombycidae), there are two sources of α-amylases, the digestive fluid and the hemolymph (Yokoyama 1959). The activity of *B. mori* α-amylase (BmAMY) was studied in both polyvoltine and bivoltine races. The polyvoltine races have adapted to tropical climate zones and exhibit high survival rate and short rearing time, although their silk fibers are short (∼500 – 700 meters) and of poor quality. Conversely, the bivoltine races have adapted to temperate zones and produce longer (1200–1500 meters) and higher quality silk fibers. However, the bivoltine races are weak and susceptible to diseases when reared in tropical zones (Murakami 1989). Interestingly, the activity of α-amylase in the digestive fluid of the polyvoltine races was higher than that of the bivoltine races, although the activity in their hemolymph did not differ. It was suggested that the increased enzyme activity of the polyvoltine races might be an adaptation to survive better in the tropical conditions (Abraham et al. 1992). Moreover, the activity of the α-amylase in digestive fluid was higher than that in the hemolymph (Abraham et al. 1992; Promboon et al. 1993). In Thai polyvoltine races, the α-amylase of the digestive fluid from the gut had an optimal pH of 9.8, whereas that of the hemolymph was 6.5 (Promboon et al. 1993). However, the function of α-amylase in hemolymph is still unclear and it has been suggested that it may be involved in the degradation of fat body glycogen (Wyatt 1967).

The *Amy* genes have been studied in many animals such as insects (Grossman et al. 1997; Da Lage *et al.* 2003; Saltzmann et al. 2006), chicken (Benkel et al. 1997), shrimp (Moal et al. 2000), oyster (Sellos et al. 2003), pig (Darnis et al. 1999), and human (Horii et al. 1987). In *B. mori,* the truncated sequence of the *BmAmy* cDNA has been registered in the National Center for Biotechnology Information (NCBI) database and its gene expression in the salivary gland has been reported (Parthasarathy and Gopinathan 2005). Since the activity of α-amylase in the digestive fluid is different between the polyvoltine and bivoltine races, the complete sequence of the gene encoding BmAMY should be studied for further elucidation of the difference. In this study, the full-length cDNA sequence, the complete genomic sequence of *BmAmy* in a polyvoltine race of Thailand, Nanglai strain and the expression of this gene are reported and it was found that this gene was expressed only in the foregut at the larval stage. Using a transgenesis technique, it was shown that the *BmAmy* promoter was active in the foregut.

## Materials and Methods

### Insects

A local Thai silkworm, the Nanglai strain, was obtained from Phuttaisong Silkworm Research Station, Thailand, and the larvae were reared on mulberry leaves. The w1-pnd strain (Uchino et al. 2006) and the *UAS—GFP* line (Kobayashi et al. 2007) were obtained from the National Institute of Agrobiological Sciences, Tsukuba, Japan, and reared on an artificial diet (Nihonnosanko, Japan) at 25° C.

### Full-length *BmAmy* cDNA sequence

Total RNA was extracted from the heads to the third segments of 5^th^ instar larvae of the Nanglai strain using TRIzol reagent (Gibco BRL, www.lifetech.com). First strand cDNA was synthesized using the Reverse Transcription System Kit (Promega, www.promega.com) according to the manufacturer's instructions. The primers (Table 1) were designed based on the truncated sequence of the *BmAmy* gene (GenBank Accession No.U07847). First strand cDNA was used as a template to amplify the *BmAmy* cDNA with the primers amyF1 and amyR1 (Table 1) under the following conditions: 95° C for 3 min; 20 cycles at 94° C for 1 min; 60° C for 30 s; 70° C for 2 min; and 70° C for 10 min. The PCR product was cloned into the pGEMT Easy plasmid vector (Promega) and sequenced at Macrogen, Korea. To obtain the full-length *BmAmy* cDNA of Nanglai, both 5′ and 3′ Rapid Amplification of cDNA Ends (RACE) were conducted. The 3′-RACE was performed using the Marathon cDNA Amplification Kit (Clontech, www.clontech.com) and the genespecific primer, amyF1 (Table 1). Amplification condition included preheating 1min at 94° C, 10 cycles of denaturing at 94° C for 30 sec, annealing at 72° C for 4 min, extending at 70° C for 4 min and 20 cycles of denaturing at 94° C for 20 sec, annealing and extending at 68° C for 4 min. The 3′-RACE PCR fragment was cloned and sequenced. The 5′-RACE was carried out using the FirstChoice RLM—RACE Kit (Ambion, www.ambion.com) and the gene-specific primers outer reverse primer and inner reverse primer (Table 1). The PCR condition was preheated at 95° C for 2 min, followed by 35 cycles of denaturing at 95° C for 30 sec, annealing at 60° C for 30 sec, extending at 68 ° C for 1 min and final extending was carried out at 68° C for 5 min. The 5′-RACE PCR fragments were cloned and sequenced.

**Table 1.**
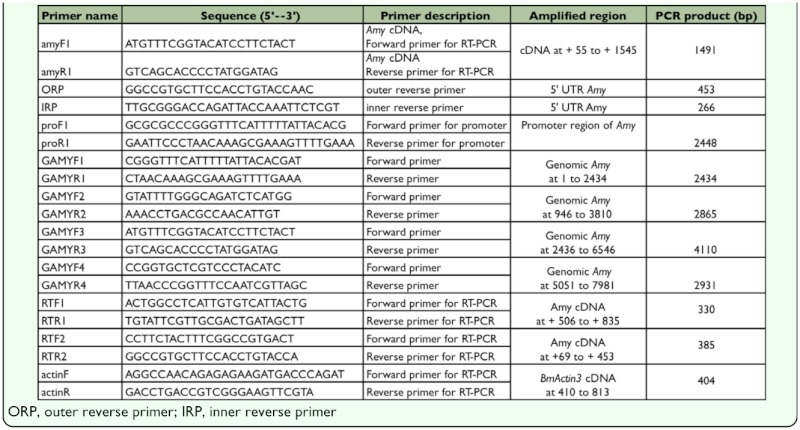
Primers used for the amplification of Nanglai *BmAmy* cDNA and the complete genomic sequence.

### Sequencing the genomic *BmAmy* gene

To identify the structure of the Nanglai *BmAmy* gene, KAIKOBLAST (http://kaikoblast.dna.affrc.go.jp/) was used to find the scaffold corresponding to the Nanglai *Amy* cDNA in the database of the silkworm genome project. Four primer sets (GAMYF 1–4 and GAMYR 1–4, Table 1) were designed for the amplification of the entire Nanglai *BmAmy* genomic region using the sequences of the scaffold and the Nanglai cDNA. Genomic DNA extracted from 1^st^ instar larvae of the Nanglai strain was used as the template for amplification with each primer set. Four obtained PCR products were cloned and sequenced. These sequences were combined together to obtain the complete genomic *Amy* gene sequence.

### Expression analysis using RT-PCR

Total RNAs were extracted with TRIzol reagent (Gibco BRL) from several tissues including the hemolymph, salivary gland, Malpighian tubule, silk gland, gut, and fat body of mid-5^th^ instar larvae of the Nanglai strain. RT-PCR High-Plus kit (Toyobo, www.toyobobiologics.com) and three primer sets, set 1 (RTF1 and RTR1), set 2 (RTF2 and RTR2) and set 3 (amyF1 and amyR1) (Table 1) were used to perform reverse transcriptase polymerase chain reactions (RT-PCR) with the extracted total RNAs. The primers actinF and actinR (Table 1) were used for RT-PCR of the *B. mori Actin3* gene (Accession No. U49854.1) as control. With the primer set 1, 2 and the primers used to amplify *Actin3,* the PCRs were performed at 95° C for 2 min, followed by 25 cycles of denaturing at 95° C for 15 sec, annealing at 64° C for 1 min and extending at 60° C for 5 min. The PCR condition used for RT-PCR with the primer set 3 was the same condition used to amplify *BmAmy* cDNA. The PCR products were analyzed on 1% agarose gels.

### Southern blotting

Southern blotting was performed using the Amersham gene images AlkPhos direct labeling detection system (Amersham, www.gelifesciences.com). Ten micrograms of the genomic DNA extracted from 5^th^ instar larvae of the Nanglai strain were digested with two restriction enzymes, *Bam*HI and *Eco*RI, at 37° C overnight and analyzed on a 0.8% agarose gel. The DNA fragments transferred from the gel were hybridized with the probe generated from the amplified product (1491 bp) of amyF1 and amyR1 (Table 1).

### Construction of the *pBac[BmAmy-GAL4, 3xP3-DsRed]*

The 2448 bp promoter region of the *BmAmy* gene was amplified using Nanglai genomic DNA as a template with the primers proF1 and proR1 (Table 1). The DNA fragment was cloned into the pTA2 vector (Toyobo) and sequenced. The *Actin3* (*A3*) promoter from *pBac[A3-GAL4, 3xP3-DsRed2]* (Imamura et al. 2003) has been replaced by Nanglai *BmAmy* promoter to form the *pBac[BmAmy-GAL4, 3xP3-DsRed]* plasmid.

### Transgenesis and screening of *B. mori*

To generate transgenic silkworms with the *pBac[BmAmy-GAL4, 3xP3-DsRed]* construct, the *pBac[BmAmy-GAL4, 3xP3-DsRed]* was co-injected with the helper plasmid *pHA3PIG* into pre-blastoderm embryos of the w1-pnd strain as described previously (Tamura et al. 2000). After injection, the G1 embryos were allowed to develop at 25° C and reared on an artificial diet (Nihonnosanko, Japan). Transgenic silkworms were screened from the G1 embryos using a fluorescence stereomicroscope (Leica, www.leica.com) equipped with the filter sets to detect DsRed expression in their stemmata. The expression of *DsRed* was controlled by the activity of the artificial promoter, 3xP3.

## Results

### Sequencing of the full length *BmAmy* cDNA of the Nanglai strain

**Figure 1.**
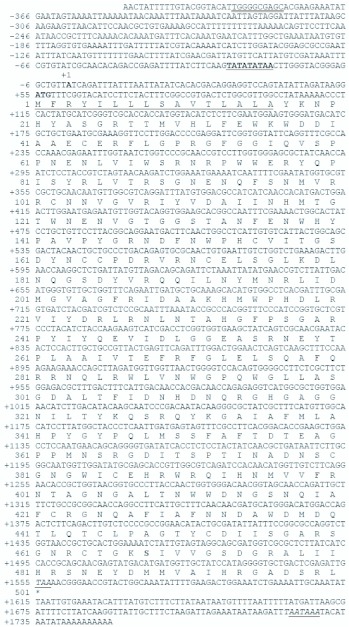
Nucleotide sequence of the *Bombyx mori* Nanglai *BmAmy* cDNA , the 5′ non-coding region and deduced amino acid sequences. The numbers with a plus sign indicate the nucleotide position from the transcription initiation site (+1). The amino acid sequence is numbered sequentially from the first amino acid, the methionine. The putative TATA box (TATATATAA) is bolded and underlined. The putative GC box is double underlined. The proposed signal peptide is underlined. The stop codon and consensus polyadenylated signal sites are in italics and underlined, respectively. High quality figures are available online.

A truncated sequence of *BmAmy* cDNA has been registered in GenBank (Accession No. U078470). According to this information, RT-PCR was performed to obtain a partial *BmAmy* cDNA sequence using primers designed from the database sequence and total RNA from the Nanglai strain. To identify the full-length Nanglai *BmAmy* cDNA, 5′ and 3′ rapid amplifications of cDNA ends (RACE) were carried out. The full-length Nanglai *BmAmy* cDNA sequence (Accession No. GQ274006) was 1749 bp long and contained a 1503 bp ORF (Figure 1). The 54 bp long 5′ untranslated region was followed by the start codon ATG at position +55 to +57. The termination codon TAA was at position +1555 to +1557 and was followed by a 192 bp 3′ untranslated region containing a polyadenylation site (AATAAA) at position +1724 to +1729 (Figure 1). The *BmAmy* open reading frame (ORF) sequence was aligned with the 738 bp ORF of the database sequence. Although the *BmAmy* ORF sequence alignment showed 99 % identity with the previous data (Accession No. U078470), the ORF sequence had three more extra regions, at positions +378 to + 674 (297 bp in length), positions +1068 to +1077 (10 bp in length) and positions +1100 to +1557 (99 bp in length). The *BmAmy* cDNA sequence encoded for a deduced protein with 500 amino acid residues. The protein contained a signal peptide spanning amino acid residues 1–16 that would be cleaved off, leaving a mature protein of 484 amino acids. The theoretical molecular weight and predicted isoelectric point of the mature BmAMY protein were 55 kDa and 8.24, respectively, when calculated using the compute pI/Mw tool of ExPASy web (http://www.expasy.ch/tools/pi-tool.html).

### Genomic structure of *BmAmy* gene

**Figure 2.**
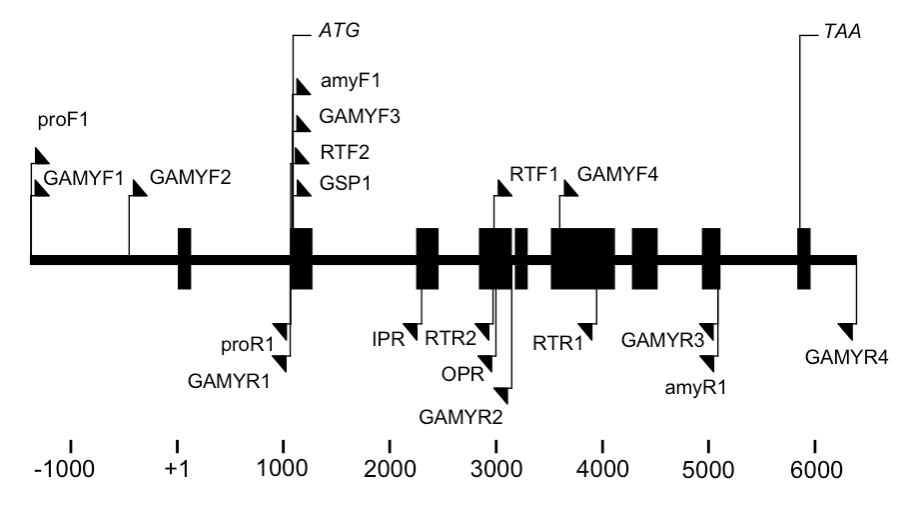
Physical map of the *Bombyx mori* Nanglai *BmAmy* gene. The closed boxes indicate exons. Arrows show the primers used to amplify the cDNA and the gene. High quality figures are available online.

Genomic DNA of the Nanglai strain was amplified with four primer sets. The PCR fragments were sequenced and combined together, revealing the 7981 bp Nanglai *BmAmy* genomic DNA sequence (Accession No. GQ274006), including 5′- and 3′-flanking regions, exons, and introns (Figure 2). The Nanglai *BmAmy* gene consisted of nine exons separated by eight introns. The first exon, the smallest, was 53 bp long and the largest exon, the sixth exon, was 509 bp long. The other exons varied in size from 99 to 201 bp. The intron size varied considerably, from 133 bp for the fourth intron to 1048 bp for the second intron. The translation start codon ATG was found in the second exon and the stop codon TAA was located in the ninth exon. The 5′-end non-coding region was 1429 bp long upstream of the transcription start site (+1). A putative consensus TATA (TATAA) box and a putative GC box were located from nucleotide position -29 to -21 and from -388 to -379 upstream of the transcription start site, respectively. Southern blot analysis was performed to estimate the copy number of the *BmAmy* gene in the Nanglai genome, using the cDNA as a probe. Genomic DNA digested with *Bam*HI showed a single fragment of 5.1 kb, whereas genomic DNA digested with *Eco*RI showed two fragments of 4.5 kb and 3.7 kb (data not shown). It is suggested that the *BmAmy* is a single-copy gene in Nanglai.

### Analysis of Nanglai BmAMY

**Figure 3.**
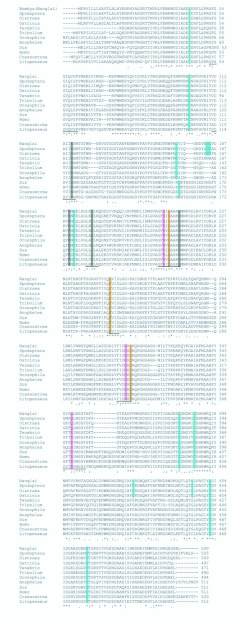
Multiple alignment of *Bombyx mori* α-amylase amino acid sequences using ClustalW version 1.81. The amino acid sequences of α-amylase were aligned to show maximum sequence identity. The seven conserved sequence regions common to all animal α-amylases are underlined. Active site residues (D209, E260, D311) are shaded in yellow. Calcium binding residues (N116, R170, D179, H213) are shaded in gray. The pink shade indicates chloride binding residues (R207, N309, Q347). Cysteine residues are shaded in blue. All amino acid sequences used for alignment were obtained from NCBI and their accession numbers are the same as in Table 2. High quality figures are available online.

Multiple alignment of the deduced amino acid sequence of Nanglai BmAMY with known sequences of α-amylases from other species was performed to identify identity percentage (Table 2) and conserved regions (Figure 3). Two aspartic acid residues (D209 and D311) and a glutamic acid (E246) residue present in the Nanglai BmAMY protein are conserved in the active sites for catalytic activity of most α-amylase protein sequences. In addition, three conserved histidine residues involved in substrate recognition were present at His117, His213, and His310 (Qian et al. 1994). Four amino acid residues, asparagine (N116), arginine (R170), aspartic acid (D179), and histidine (H213) comprised a putative calcium-binding site. Moreover, three amino acid residues, arginine (R207), asparagine (N309), and glutamine (Q347) involved in chloride binding were found and were needed for full catalytic activity. The latter amino acid residue, Q347, was not found in the α-amylase proteins of other animals, and instead, an arginine (R) residue was present at this site (Strobl et al. 1997; 1998). Twelve cysteine residues were found at positions 44, 102, 155, 164, 165, 172, 380, 386, 422, 445, 452, and 464 (Figure 3). Eight of these 12 cysteine residues (positions 44, 102, 155, 172, 380, 386, 452, and 464) were conserved in all animal α-amylase proteins and formed four disulfide bridges (Janacek 1993). The two cysteine residues at positions 422 and 445 may form another disulfide bridge, as was suggested for *Penaeus vannamei* and *Aedes aegypti,* which also have two cysteines at the same positions (Grossman and James 1993; Van Wormhoudt and Sellos 1996). The remaining pair at position 164 and 165 has been found only in some species of Lepidoptera but not in other animals. The different number of disulfide bridges may be related to differences in enzyme activity (Da Lage et al. 1996). Finally, the seven conserved motifs found in all animal α-amylase proteins were also present in Nanglai BmAMY (Figure 3).

**Table 2.**
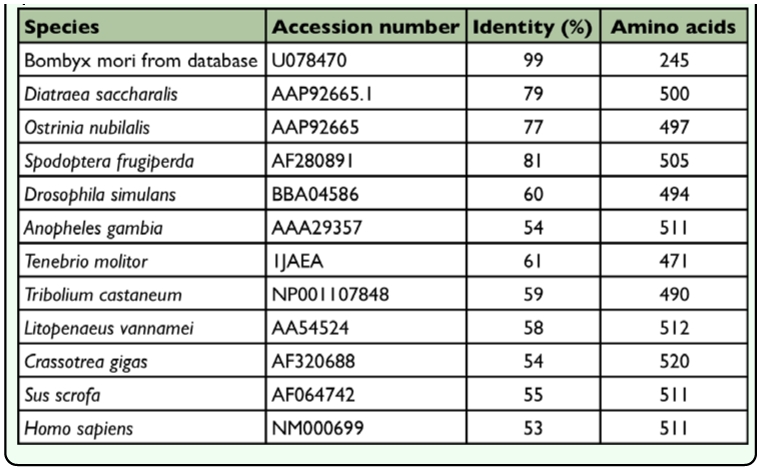
Pair-wise ClustalW analysis using the deduced amino acid sequences of Nanglai BmAMY compared with AMYs from other known species that are vailable in the NCBI database.

**Figure 4.**
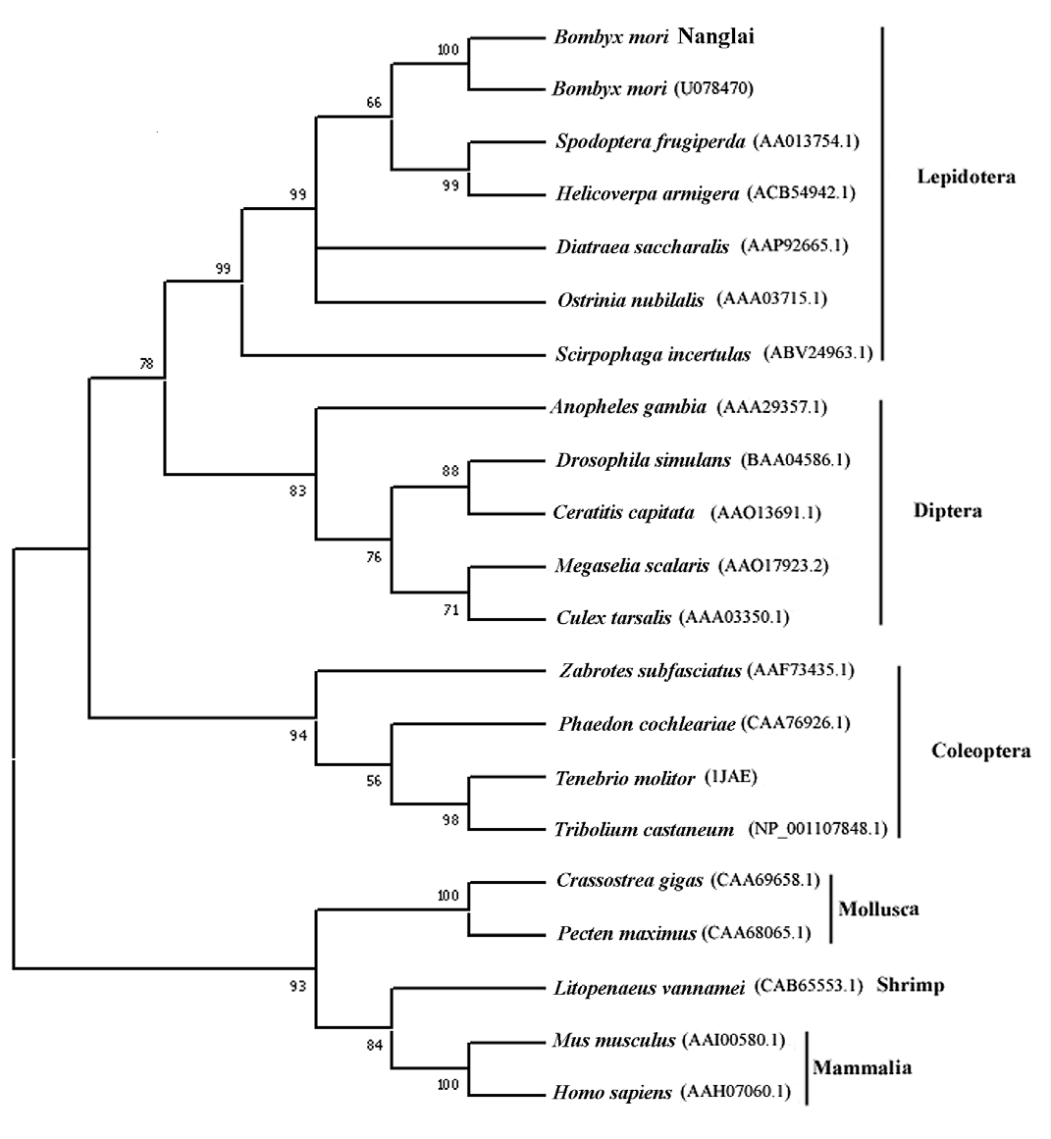
Phylogenese analysis of *Bombyx mon* Nanglai BmAMY with α-amylases of some other species. The tree based on their amino acid sequences was constructed by MEGA 3.1. The numbers at the nodes are the percentage of 1000 bootstrap re-samplings. The accession number of each species is shown in parentheses. High quality figures are available online.

A phylogenetic tree of α-amylase was constructed with the deduced amino acid sequences from some animal species using *MEGA* version 3.1 (Kumar et al. 2004). The tree clearly showed four clades of the orders Lepidoptera, Diptera, Coleoptera and of the non-insects (Mollusca, Shrimp and Mammalia) (Figure 4). As expected, the tree grouped *B. mori,* Nanglai strain, into the Lepidoptera clade. Therefore, the α-amylase amino acid sequence can be applicable for the classification of insect order.

### Tissue specificity of the *BmAmy* gene

**Figure 5.**
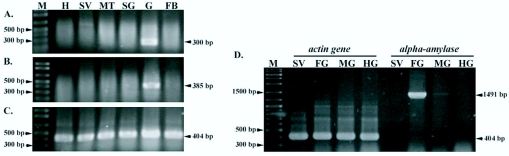
Tissue-specific expression of the *Bombyx mori* Nanglai *BmAmy* gene was analyzed using RT-PCR with primer sets 1 (A), 2 (B), and 3 (D). RT-PCR of the *Bombyx mori actin3* gene is shown in C and D. Total RNAs extracted from the hemolymph (H), salivary gland (SV), Malpighian tube (MT), silk gland (SG), gut (G), fat body (FB), foregut (FG), midgut (MG), and hindgut (HG) of the Nanglai strain were used as templates for RT-PCRs. Lane M shows 100-bp and 1-kb DNA ladders as size markers. High quality figures are available online.

To elucidate the tissue specificity of *BmAmy* gene, RT-PCRs were performed using total RNAs extracted from several tissues of the 5^th^ instar larvae Nanglai strain at the 4^th^ to 5^th^ day. Three sets of primers, RTF1 and RTR1; RTF2 and RTR2; and amyF1 and amyR1, were used to amplify three different regions of the Nanglai *BmAmy* gene and three DNA fragments, 330 bp, 385 bp, and 1491bp, were obtained respectively. The results showed that the expression of *BmAmy* was found only in the gut (Figure 5A and B). Then, the gut was separated into three parts, the foregut, midgut, and hindgut, and the RT-PCRs were repeated. The result showed that gene expression was limited to the foregut (Figure 5D).

**Figure 6.**
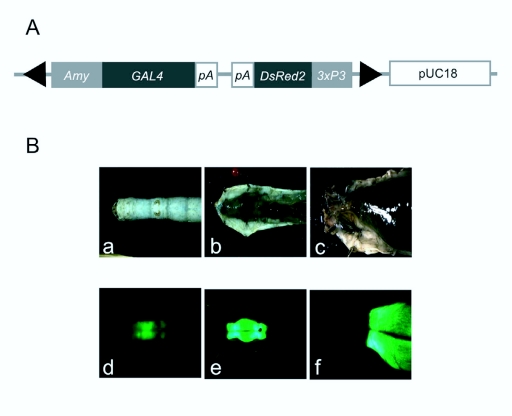
Characterization of the promoter activity of the *Bombyx mori* Nanglai *BmAmy* gene. A: physical map of the vector *pBac[Amy-GAL4 3xP3DsRed].* B: Localization of EGFP expression in the transgenic silkworm possessing the *Amy-GAL4* and *UAS-EGFP* constructs. Bright-field (a–c) and fluorescence images (d–f) of the 5^th^ instar larvae at the 5^th^ day are shown. Living larvae (a and d) and dissected larvae at low (b and e) and higher (c and f) magnifications are shown. High quality figures are available online.

To investigate the promoter activity of *BmAmy* gene, a vector of the *GAL4* gene with the 5′-flanking region of the *Amy* gene was constructed (Figure 6A), and transgenic silkworm with the *pBac[BmAmy-GAL4, 3xP3-DsRed]* construct was generated (Table 3).

**Table 3.**
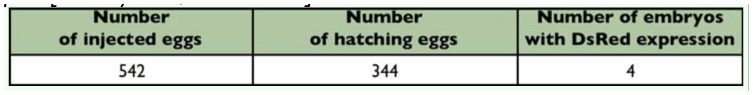
Transenesis and screening of silkworms containing *pBac[BmAmy-Gal4, 3xP3-DsRed]* vector

The transgenic silkworm was crossed with the *UAS—EGFP* line and the expression of EGFP in the progeny was observed. It was found that the EGFP was expressed only in the progeny's foregut (Figure 6B). Therefore, the result was in agreement with those obtained from the RT-PCR experiment and it is likely that the *BmAmy* gene isolated in this study is expressed only in the foregut and that BmAMY is secreted into the digestive fluid.

## Discussion

The full-length *BmAmy* cDNA and its genomic structure in the Thai polyvoltine silkworm, Nanglai strain, have been successfully identified. This is the first report of the complete nucleotide sequence of the *BmAmy* gene in *B. mori.* The genomic sequence of *BmAmy* is approximately 7.9 kb long and contains nine exons, which is quite large compared with that of other insects, including *Drosophila melanogaster* (2.5 kb; Boer and Hickey 1987), *Aedes aegypti* (3.2 kb; Grossman et al. 1997), *Spodoptera frugiperda* (5.0 kb; Da Lage et al. 2002), *Ceratitis capitata* (2.3 kb. Da Lage et al. 2002), *Tribolium castaneum* (5.1 kb; Abukashawa et al. unpublished). The number of introns within the *Amy* genes of insects varies considerably. In the genus *Drosophila,* five of 146 species contain genes with no introns and the others have only one intron (Da Large et al. 1996). The *Amy* genes of *Apis mellifera* (Ohashi et al. 1999) and *Ceratitis capitata* (Da Lage et al. 2002) contain only two introns and that of *Tribolium castaneum* (Abukashawa et al. unpublished) has three introns. Conversely, the *Amy* genes of Lepidoptera species generally contain a large number of introns (Sellos and Wormhoudt 2002). For example, *Spodoptera frugiperda* has six introns and *Ostrinia nubilalis* has seven introns (unpublished data from NCBI). The *BmAmy* gene obtained in this study has eight introns.

In *B. mori,* BmAMY is present in the digestive fluid and hemolymph (Yokoyama 1959; Promboon et al. 1993). The molecular weight of the enzyme purified from the digestive fluid of polyvoltine strains has been reported to be 55 kDa (Kanakatsu 1978). The molecular weight of the protein estimated from the gene sequence obtained in this study is 55 kDa. Therefore, it is likely that the *BmAmy* gene isolated in this study encodes the BmAMY enzyme found in digestive fluid. However, the molecular weight of α-amylase in hemolymph has not been reported. A comparison of the deduced amino acid sequence of the BmAMY with those of other insects reveal that the amino acid sequence of the BmAMY is 78, 79, and 81% identical to that of three species of Lepidoptera: *Ostrinia nubilalis* (AAA03715.1; Foster et al. unpublished), *Diatraea* saccharalis (AAP92665.1; Guerra et al. unpublished), and *Spodoptera frugiperda* (AF280891_1; Da Lage et al. 2002), respectively. In addition, it is more than 50% identical to that of other animals such as human, pig, oyster, shrimp, fruit fly, and mosquito. This homology clearly indicates that these proteins are of the same enzyme family. Moreover, when compared with α-amylase proteins of other animals, the amino acid sequence of BmAMY from Nanglai strain is highly conserved at the active sites and calcium binding sites.

The *BmAmy* encodes a chloride-dependent α-amylase that requires chloride for full activity (Janacek 1997; Qian et al. 2005). The three amino acid residues involved in the chloride binding of most animal α-amylases include two arginine (R) residues and an asparagine (N) residue. Conversely, *BmAmy* differs from those of other animals with the exception of Lepidoptera, in that one of the arginines is replaced with glutamine (Q347). The amino acid sequences of the chloride-binding sites in Lepidoptera are identical (Strobl et al. 1997). The seven conserved motifs that are commonly found in other animal α-amylases are also present in the deduced BmAMY protein isolated from Nanglai strain (Svensson 1988; Janacek 1997).

In insects, *Amy* is expressed in different tissues. For example, in *Drosophila ananassae* (Da Lage et al. 2003) the *Amy* gene is expressed in the gut. Similarly, *Amy* is expressed in the crop and midgut of *Lutzomyia longipalpis* (Hill and Orchard 2005). In *Blattella germanica,* the tergal glands are specialized exocrine glands that secrete α-amylase (Saltzmann et al. 2006). In *Ae. aegypti, Amyl* is expressed only in salivary glands (Grossman and James 1993). In *B. mori,* whole-mount *in situ* hybridization reveals that *Amy* is expressed in the salivary glands (Parthasarathy and Gopinathan 2005). However, our results indicate that the *BmAmy* is expressed mainly in the foregut of a polyvoltine race, and the promoter activity of the gene supports this finding. Therefore, it is likely that the *BmAmy* gene isolated from Nanglai, a polyvoltine race, is expressed mainly in the gut and that the product is released to the digestive fluid. Two loci of *Amy* gene, *Amy-d* and *Amy-h,* have been reported to exist in *B. mori* (Matsumura 1933). Both loci are closely located on the chromosome 8. Null mutant of *Amy-d* is lacking amylase activity in the digestive juice (Kanekastu 1972) while in the null mutant of *Amy-h,* the α-amylase activity in the hemolymph is very weak (Tanaka et al. 1976).

In the digestive juice, there are 5 isoforms of α-amylase and all these isoforms have the same molecular weight (Asakawa and Hamano 1989). Therefore, it is likely that α-amylase in the digestive juice is encoded by a single gene. Our results consistently indicate that the *BmAmy* gene identified in this study is present as a single copy in the Nanglai strain. Moreover, by searching the genomic database of *B. mori* for *Amy* homologs, no *Amy* homolog was found. This suggests that the *Amy* identified in this study may be that of *Amy-d* locus. However, if it is ture that no *Amy* homolog exists and BmAMY is encoded by a single locus, the question arise why the α-amylase activity in the digestive fluid is different from that in the hemolymph. At present, it is postulated that the *BmAmy* gene encodes the native proteins in the gut, and the enzyme is released into hemolymph and digestive fluid. In the different environments of these regions, the enzyme may be subjected to different post-translational modifications that are responsible for the differences in the activity of the enzyme from the two sources.

## Abbreviations

*Amy*,: *α*-amylase gene;
BmAMY,: *Bombyx mori α*-amylase enzyme
